# Root resorption caused by aligners, self-ligating appliances, and conventional fixed appliances: a CBCT-based meta-analysis

**DOI:** 10.1186/s12903-025-06639-2

**Published:** 2025-07-26

**Authors:** Patrik Kreuter, Kata Sára Haba, Szilvia Kiss-Dala, Petrana Martineková, Emese Ábrám, Andrea Bródy, Dániel Végh, Péter Hegyi, Noémi Katinka Rózsa, Dorottya Bányai

**Affiliations:** 1 Department of Paediatric Dentistry and Orthodontics, Semmelweis University, Budapest, Hungary; 2 Centre for Translational Medicine, Semmelweis University, Budapest, Hungary; 3 Department of Prosthodontics, Semmelweis University, Budapest, Hungary; 4 Institute for Translational Medicine, University of Pecs, Pécs, Hungary; 5 Department of Oral Diagnostics, Semmelweis University, Budapest, Hungary; 6 Institute of Pancreatic Diseases, Semmelweis University, Budapest, Hungary

**Keywords:** CBCT, Root resorption, Aligner, Fixed appliances, Self-ligating

## Abstract

**Background:**

Orthodontically induced inflammatory root resorption (OIIRR) is a common adverse effect of orthodontic treatments. Radiographs are routinely used to diagnose OIIRR; however, 3-dimensional cone beam computed tomography (CBCT) studies have recently been conducted to assess hard tissue loss more accurately. There is controversial evidence of differences between aligners and fixed appliances in terms of OIIRR. This meta-analysis aims to investigate the differences in OIIRR between fixed appliances and aligners based on recent CBCT-based studies.

**Methods:**

A systematic review and meta-analysis was conducted after PROSPERO registration. Four databases (MEDLINE, Embase, CENTRAL, Scopus) were systematically screened to identify studies reporting on (P) patients with full permanent dentition treated with (I) aligners or (C) fixed orthodontic appliances that reported on (O) root resorption detected by CBCT, without any date or language restrictions. Exclusion criteria included incomplete dentition, root canal treatment, dental trauma, previous root resorption, and developmental abnormalities. Means and mean differences were used as effect size measures, Chi-squared tests for subgroup differences, and I^2^ values for heterogeneity were calculated. Risk of bias was evaluated using ROBINS-I and RoB2 tools.

**Results:**

The meta-analysis included five studies with 334 participants. Data on upper incisors were sufficient for analysis. Differences in OIIRR between aligners and fixed appliances did not reach statistical significance (*p* > 0.05), and neither group presented clinically relevant OIIRR (< 1 mm). A moderate to high risk of bias was present.

**Discussion:**

All treatment modalities caused similar, clinically irrelevant levels of OIIRR in the investigated population. The treatment modality should be selected based on biomechanics, expected outcomes, and individual preferences. Clinicians should not prioritize aligners over fixed appliances in the non-risk population in fear of OIIRR. The results should be interpreted cautiously due to the risk of bias and heterogeneity.

**Registration:**

PROSPERO: CRD42023481411.

**Supplementary Information:**

The online version contains supplementary material available at 10.1186/s12903-025-06639-2.

## Background

The standard care for orthodontic patients with permanent dentition traditionally includes using fixed appliances, which often cause esthetic and hygienic difficulties during treatment [1]. Since the early 2000s, there have been ongoing efforts to develop invisible orthodontic appliances (aligners) to become a reliable treatment option to replace fixed appliances [2]. As aligners offer more comfortable and esthetic treatment with less associated periodontal risk and higher levels of patient satisfaction, the demand for these treatments is growing rapidly [3, 4]. On the other hand, there is controversial evidence on the differences in treatment outcomes between the two interventions [5, 6] and adverse effects, such as orthodontically induced inflammatory root resorption (OIIRR) [7, 8], which is reportedly also present in aligner treatments [6]. 

OIIRR is an irreversible adverse effect that results in the loss of external hard tissue of the dental roots, affecting up to 90% of orthodontic patients, depending on the population studied [9]. As the terminology suggests, resorption is the result of a sterile inflammatory process mediated by activation of macrophages and macrophage-like cells triggered by the orthodontic forces applied [10]. In most cases, it does not reach clinically significant levels and can be observed as a blunted apex on radiographs. In contrast, severe cases compromise the longevity of the affected teeth and jeopardize further orthodontic treatments [9]. 

Known risk factors of OIIRR include a history of dental trauma, previous root resorption, prolonged orthodontic treatment, and genetic predisposition. Individual characteristics, such as age, sex, systemic factors (i.e., hormonal anomalies), malocclusion, root shapes and deformities, root position relative to the alveolar bone, bone density, history of endodontic treatment, direction and magnitude of tooth movement may also indicate different probabilities of root resorption, but there are contradictions in the literature [9–14]. 

Intraoral and panoramic radiographs are routinely used to detect root resorption by comparing pre-, mid-, and post-treatment root lengths. However, previous findings suggest that 2-dimensional imaging techniques are unreliable in accurately assessing root resorption due to difficulties of landmark identification, distortion, and overlapping of structures [15]. As more accurate cone-beam computed tomography (CBCT) studies providing 3D data of the roots have only recently become available, the demand for revising previous findings based on radiographs has risen. When comparing the two treatment modalities, some studies claim that aligners may cause less severe root resorption [7, 16, 17], on the other hand, findings support, that these differences are negligible [8] or clinically insignificant if present [18]. There is no comprehensive guidance for clinicians to choose between the available treatments to minimize OIIRR. Therefore, our aim is to investigate the differences in root resorption between aligners and fixed appliances by comparing the latest results of CBCT-based studies.

## Methods

We report our systematic review and meta-analysis based on the recommendations of the PRISMA (Preferred Reporting Items for Systematic reviews and Meta-Analyses) 2020 Guideline [19] (Supplementary Table 1), and the Cochrane Handbook [20]. The study protocol was registered on PROSPERO (registration number: CRD42023481411). Protocol amendment was necessary throughout the systematic search, as several relevant articles measured the total tooth length reduction rather than the root length changes directly. Studies measuring both tooth length and root length were included, considering possible differences due to changes in clinical crowns.

### Eligibility criteria

Studies reporting on (population) patients with full permanent dentition (except for third molars) treated with (intervention) aligners or (comparison) fixed orthodontic appliances were included if they provided data on the change in tooth or root length in millimeters (outcome), measured on CBCT images (primary outcome) or radiographs (secondary outcome). Pooling of 3D and 2D measurements was avoided to prevent bias from the reliability differences discussed prior. Patients with a history of root canal treatment, dental trauma, developmental issues and previous root resorption and interventions, which used combined treatment (fixed appliances with aligners or orthodontic mini-implants) were excluded. Randomized controlled trials (RCTs), prospective or retrospective cohorts were included, whereas case controls, case series, case reports, surveys, review articles, and letters were excluded.

### Information sources

Our systematic search was conducted in four databases, namely MEDLINE (via Pubmed), Embase, CENTRAL (The Cochrane Central Register of Controlled Trials), and Scopus on November 13, 2023.

### Search strategy

The search key comprised two domains: (1) aligners and (2) fixed appliances or their synonyms and was adapted for each database (see Supplementary Document 1).

### Selection process

Selection was performed by two independent authors (PK and KSH) after duplicates were removed in EndNote 21 [21]. The Rayyan platform [22] was used for title and abstract selection of duplicate free results. The two reviewers performed the final selection based on full texts of remaining results. Conflicts from both selections were resolved by a third reviewer (PM). Citation chasing was performed for references and citations of the full texts included using Citationchaser [23]. 

### Data collection process

A standardized data collection sheet was created in Excel [24] based on the consensus of methodological and clinical experts. Two authors (PK and KSH) independently collected data from the eligible articles. For data available as graphs or figures, WebPlotDigitizer [25] was used for data extraction. In case of data discrepancies, authors were contacted for clarification.

### Data items

The following data were extracted from eligible articles: first author, year of publication, study population (age, sex, baseline orthodontic discrepancies), study period, type of intervention and control (including treatment duration), outcomes (means of root or tooth length changes and related standard deviation values).

### Study risk of bias assessment

Risk of bias was assessed based on the recommendations of the Cochrane Collaboration. The assessment was performed by two independent reviewers (PK, KSH). The Risk Of Bias In Non-randomized Studies - of Interventions (ROBINS-I) and the revised Cochrane risk of bias tool for randomized trials (RoB2) were used for the retrospective cohort studies and RCTs, respectively.

### Synthesis methods

Statistical analysis was conducted with R (version 4.4.3) [26], using the meta (version 8.0.2) [27] package for basic meta-analysis calculations and plots and the metafor package (version 4.8.0) [28] for multivariate models. As considerable between-study heterogeneity was assumed in all cases, a random effects model was used to pool effect sizes.

In our first approach, means were used as effect size measures with 95% confidence intervals (CI), and aligners were compared to fixed appliances, as well as self-ligating appliances (SL) to conventional fixed appliances (CFA) as subgroups. First, we used the metamean function of the meta R package. The results were visualized as forest plots. Tests of individual coefficients and confidence intervals were based on a t-distribution. Between-study heterogeneity was described by Higgins&Thompson’s I^2^ statistic [29]. Publication bias was assessed by visual inspection of Funnel plots, and Egger’s test, if at least 10 studies were available. Subgroup differences were tested using the Chi-squared test. As studies contributed data to more than one subgroup, and although the results of different subgroups corresponded to different populations, the random effects corresponding to subgroups of the same study may be correlated. To account for these possible between-study correlations, we also fitted multivariable/multilevel, setting the within-study correlation to a compound symmetric structure, using the rma.mv function from the metafor R package [28]. 

As a second approach, studies including two arms were pooled using mean difference (MD) as effect size measures with 95% CI. We used the metacont function of the meta R package; the settings were as described above. If multiple entries were extracted from the same study, the rma.mv function was used to determine the p-value of the pooled effect.

Due to the insufficient amount of data available, the analysis of secondary outcomes was not possible.

Results were considered statistically significant if pooled CIs did not contain the null effect value.

## Results

### Search and selection

The systematic search identified 6669 articles. After duplicate removal, 3956 texts were screened by title, abstract and full text, leaving 6 articles eligible for the systematic review, 5 of which were eligible for statistical analysis within a meta-analysis. An almost perfect agreement was present in the interrater reliability assessment with a Cohen’s Kappa of 0.83 for title and abstract and 0.92 for full-text selection.

**Fig. 1:**
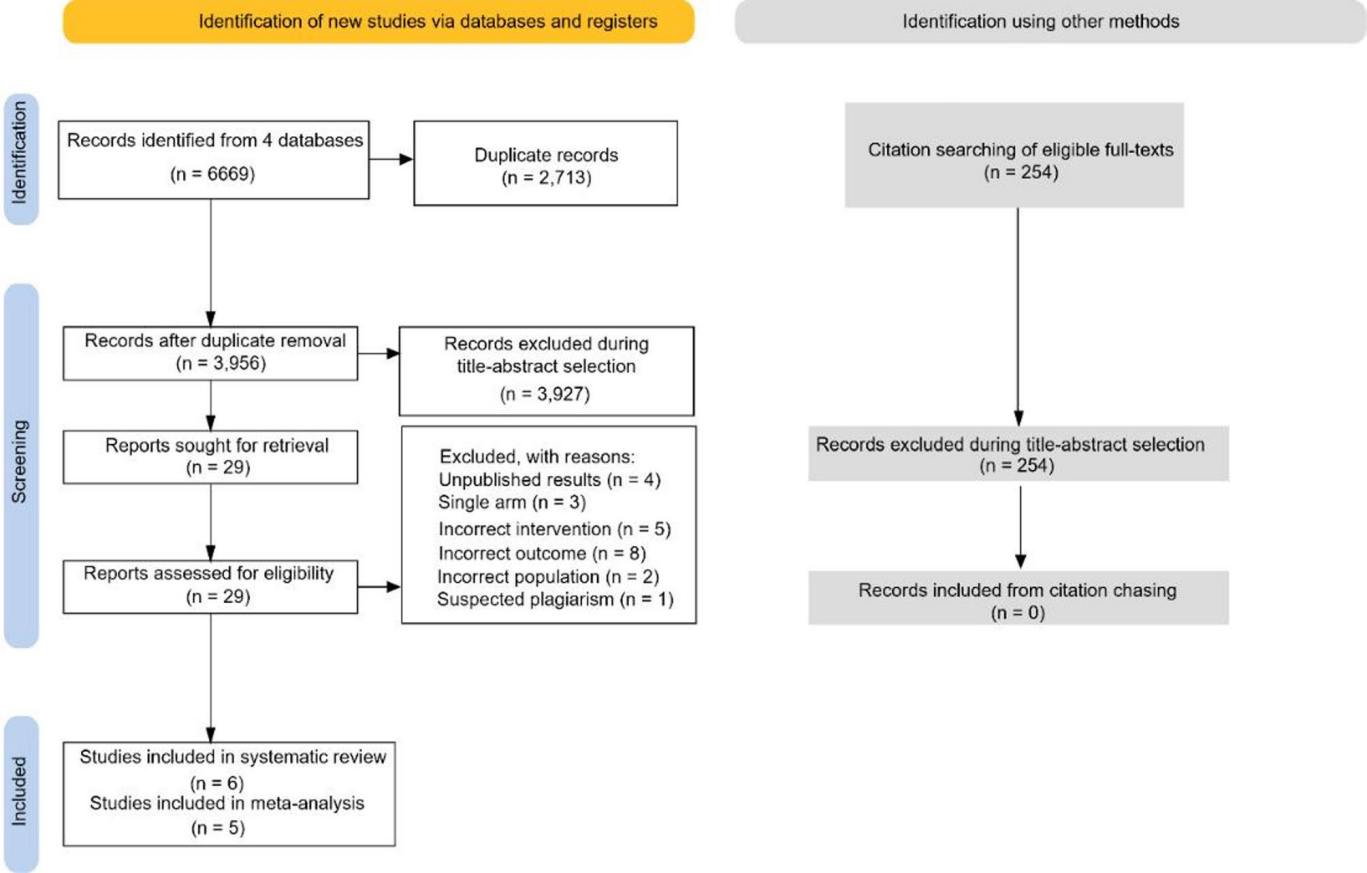
PRISMA selection flow-chart

Backward and forward citation chasing did not result in any new eligible hits. The flowchart of the selection process with the rationale for full-text exclusions is visualized in Fig. 1. Details of the full texts excluded are presented in Supplementary Table 2.

### Basic characteristics of studies included

Five retrospective studies used CBCT measurements, and one RCT used radiographs, leading to a meta-analysis applicable only to CBCT findings [7, 8, 16, 17, 30, 31]. There were differences between measurement methods, as studies measured total tooth length (incisal edge/cusp to apex) or root length (cementoenamel junction to apex), and the tooth types investigated were inconsistent—only upper central and lateral incisor measurements provided sufficient data for quantitative analysis. Data on teeth from different sides were combined from the article of Eissa et al. [17], as all other articles provided pooled measurements of both sides. An additional subgroup analysis of the conventional fixed appliance (CFA) and self-ligating fixed appliance (SL) was also performed. Measurement methods, study designs, and patient baseline characteristics are presented in Table 1, with additional raw data in Supplementary Table 3.

**Table 1:** Characteristics of studies included. C: control group; CBCT: cone beam computed tomography; I: intervention group; IO: intraoral; NA: not available; RCT: randomized clinical trial

Art. No.	Author (year, country)	Study design	No. of patients (% of females)	Angle class of malocclusion	Cases with extraction(s)	Intervention and control(s)	Outcome measurement	
1	Chen, H., et al. (2023, China)	Retrospective cohort	59 (61%)	Class II division 2	none	I: aligner (Invisalign)	CBCT tooth length of upper central incisors	
						C: conventional fixed appliance (3 M)		
						C: self-ligating fixed appliance (Damon)		
2	Eissa, O., et al. (2018, Egypt)	Retrospective cohort	33 (55%)	Class I	n/a	I: aligner (Invisalign)	CBCT tooth length of upper central and lateral incisor	
						C: conventional fixed appliance (3 M)		
						C: self-ligating fixed appliance (Damon)		
3	Li, Y., et al. (2020, China)	Retrospective cohort	70 (70%)	NA	present	I: aligner (Invisalign)	CBCT tooth length of upper and lower front teeth (canine to canine)	
						C: conventional fixed appliance (3 M)		
4	Almagrami, I., et al. (2023, China)	Retrospective cohort	40 (NA)	NA	none	I: aligner (Invisalign)	CBCT root length of upper central and lateral incisor	
						C: conventional fixed appliance (3 M)		
5	Mai, T., et al. (2021, China)	Retrospective cohort	93 (46%)	Class I, II, III	none	I: aligner (NA)	CBCT root length of upper and lower front teeth (canine to canine)	
						C: self-ligating fixed appliance (NA)		
6	Toyokawa-S., K.C., et al. (2021, Brazil)	RCT	39 (35%)	Class I	none	I: aligner (Invisalign)	IO radiograph tooth length of upper and lower incisors	
						C: conventional fixed appliance (3 M)		

### Root resorption of upper central incisors

**Fig. 2:**
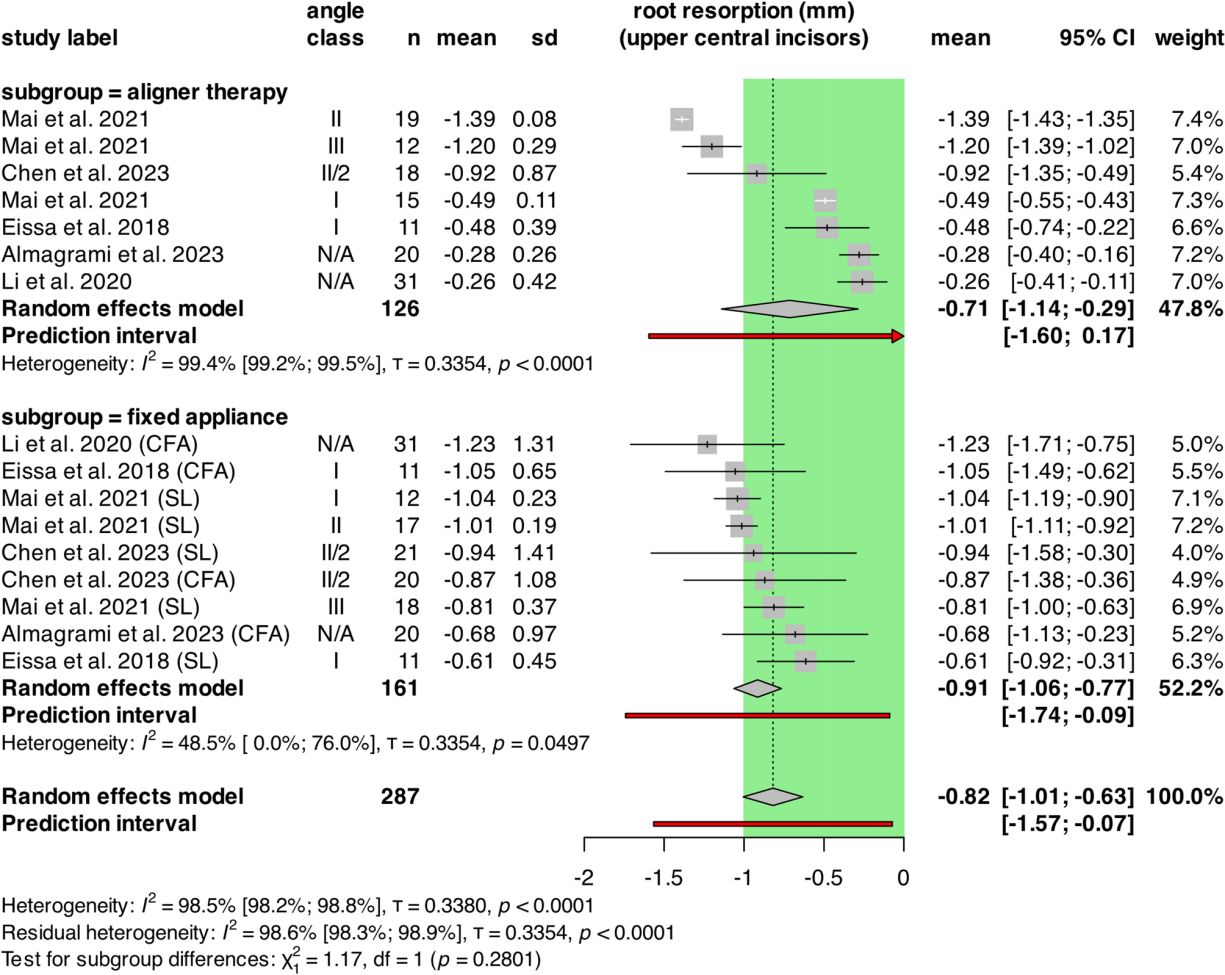
Forest plot showing OIIRR of upper central incisors in aligners versus fixed appliances. The green area represents the cut-off value of clinically not relevant extent of root resorption. CI: confidence intervals; n: number of patients; SD: standard deviation

A total of 287 patients from 5 retrospective cohort studies were included in the analysis. (Fig. 2) Mean values for length changes in upper central incisors (UCI) were − 0.71 mm [CI: -1.14; -0.29], and − 0.91 mm [CI: -1.06; -0.77] for aligners and fixed appliances (FA), respectively. Significant heterogeneity was observed in the aligner group with I^2^ = 99.4% [CI: 99.2%; 99.5%, τ = 0.3354, *p* < 0.0001] and marginally significant in the FA group with I^2^ = 48.5% [CI: 0.0%; 76.0%, τ = 0.3354, *p* = 0.0497].

The Chi-squared test for subgroup differences showed no significant difference between the aligner and FA group (*p* = 0.2801).

The random effects model resulted in -0.82 mm [CI: -1.01; -0.63] overall mean resorption. Overall heterogeneity was significant, with I^2^ = 98.5% [CI: 98.2%; 98.8%, τ = 0.3380, *p* < 0.0001].

The multilevel/multivariate model showed similar effects: mean resorption in aligner therapy was − 0.68 mm, and − 0.89 mm in the fixed appliance group. The difference was not significant (*p* = 0.2422).

For the pairwise analysis, MDs were plotted. (Supplementary Fig. 1.) The MD between aligners and fixed appliances was 0.19 mm [CI: -0.17; 0.55]. Significant heterogeneity was detected: I^2^ = 94.7% [CI: 91.9%; 96.5%, τ = 0.4364, *p* < 0.0001].

To assess the significance of the pooled effect, we carried out a multilevel/multivariate model, which indicated no significant difference between the two groups (*p* = 0.2378).

**Fig. 3:**
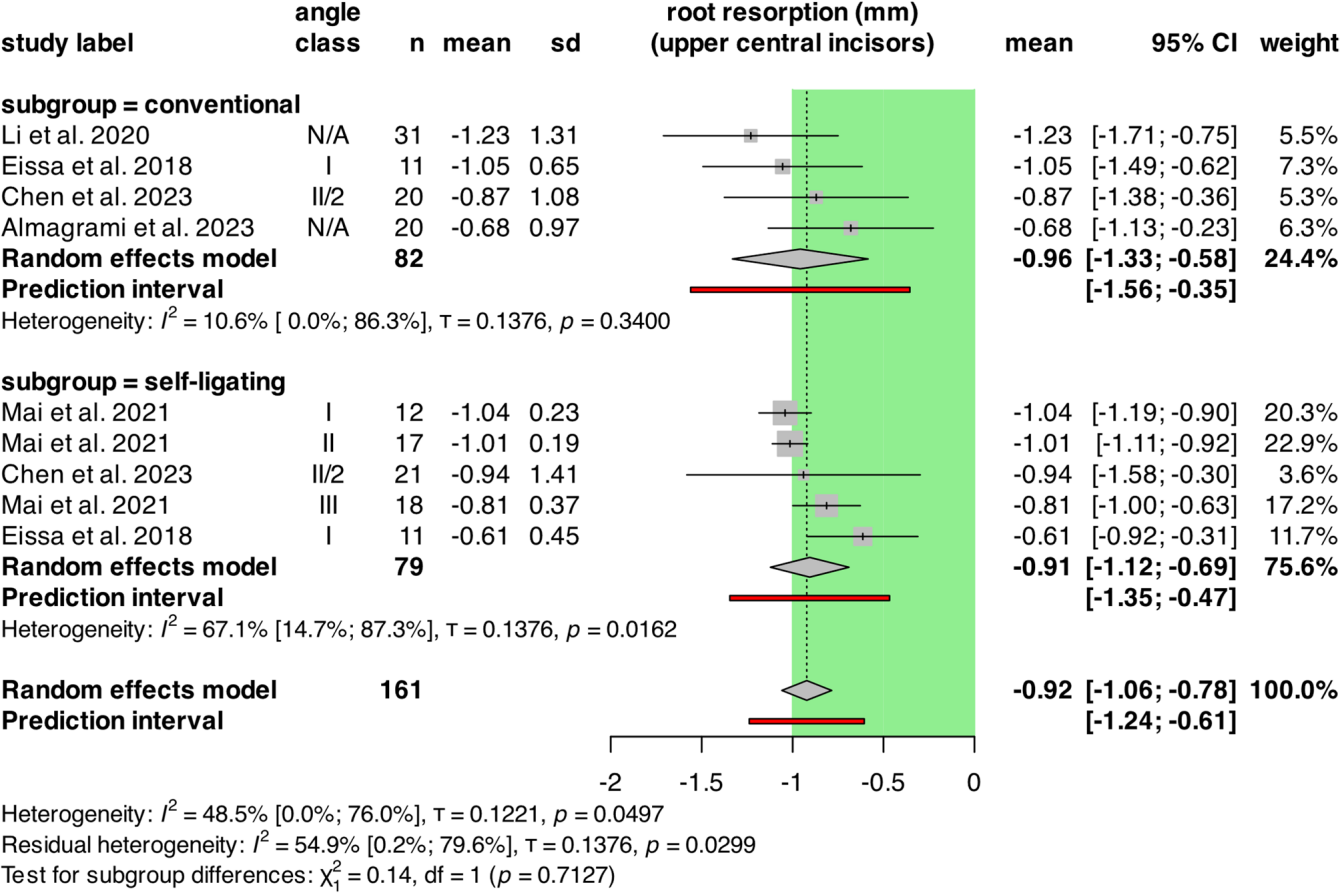
Forest plot showing OIIRR of upper central incisors in conventional versus self-ligating groups. The green area represents the cut-off value of clinically not relevant extent of root resorption. CI: confidence intervals; n: number of patients; SD: standard deviation

Another subgroup analysis was conducted to examine the differences between CFA and SL groups. (Fig. 3)

The analysis included 161 patients from 5 retrospective cohort studies. The CFA group had mean root resorptions of -0.96 mm [CI: -1.33; -0.58], the SL group had a mean value of -0.91 mm [CI: -1.12; -0.69]. Intragroup heterogeneity was not significant in the CFA group, as the I^2^ value was 10.6% [CI: 0.0%; 86.3%, τ = 0.1376, *p* = 0.3400], it was moderately significant in the SL group 67.1% [CI: 14.7%; 87.3%, τ = 0.1376, *p* = 0.0162].

There was no statistically significant difference between the groups (*p* = 0.7127).

The random effects model resulted in -0.92 mm [CI: -1.06; -0.78] overall mean resorption. Overall heterogeneity was marginally significant, with I^2^ = 48.5% [CI: 0.0%,76.0%; τ = 0.1221, *p* = 0.0497].

The multilevel/multivariate model showed similar effects: mean resorption in the conventional group was − 0.98 mm, while − 0.87 mm in the self-ligating group and the difference was not significant (*p* = 0.4889).

For the pairwise analysis, only two studies could be included with 63 patients overall. (Supplementary Fig. 2.) The MD between SL and CFA was 0.28 mm [CI: -2.74; 3.30]. The heterogeneity could not be detected due to the low number of studies.

The sigificance of the pooled effect size was: *p* = 0.4488, indicating no statistically significant difference between the two groups.

### Root resorption of upper lateral incisors

**Fig. 4:**
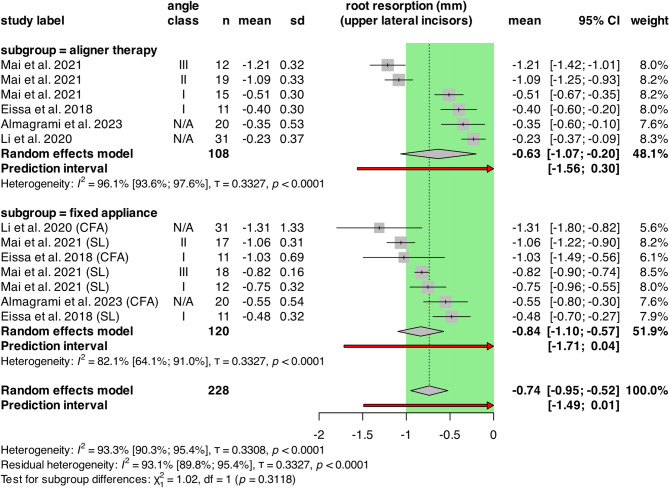
Forest plot showing OIIRR of upper lateral incisors in aligners versus fixed appliances. The green area represents the cut-off value of clinically not relevant extent of root resorption. CI: confidence intervals; n: number of patients; SD: standard deviation

Four retrospective cohort studies were included with a total population of 228 patients. (Fig. 4) The mean values for length changes in upper lateral incisors (ULI) were − 0.63 mm [CI: -1.07; -0.20] for the aligner and − 0.84 mm [CI: -1.10; -0.57] for the FA groups. The heterogeneity was significant in both groups, as the I^2^ was 96.1% [CI: 93.6%; 97.6%; τ = 0.3327, *p* < 0.0001] for the aligner group and 82.1% [CI: 64.1%; 91.0%; τ = 0.3327, *p* < 0.0001] for the FA group.

There was no statistically significant difference between the groups (*p* = 0.3118).

The random effects model resulted in -0.74 mm [CI: -0.95; -0.52] overall mean resorption. Overall heterogeneity was significant, with I^2^ = 93.3% [CI: 90.3%,95.4%; τ = 0.3308, *p* < 0.0001].

The multilevel/multivariate model showed similar effects: mean resorption in the aligner group was − 0.59 mm, while − 0.80 mm in the fixed appliance group and the difference was not significant (*p* = 0.2548).

For the pairwise analysis, MDs were plotted again. (Supplementary Fig. 3.) The MD between aligners and fixed appliances was 0.22 mm [CI: -0.20; 0.65]. Significant heterogeneity was detected: I^2^ = 87.3% [CI: 76.1%; 93.2%, τ = 0.4172, *p* < 0.0001].

No statistically significant difference was present between the two groups based on the multilevel multivariate model (*p* = 0.1870).

**Fig. 5:**
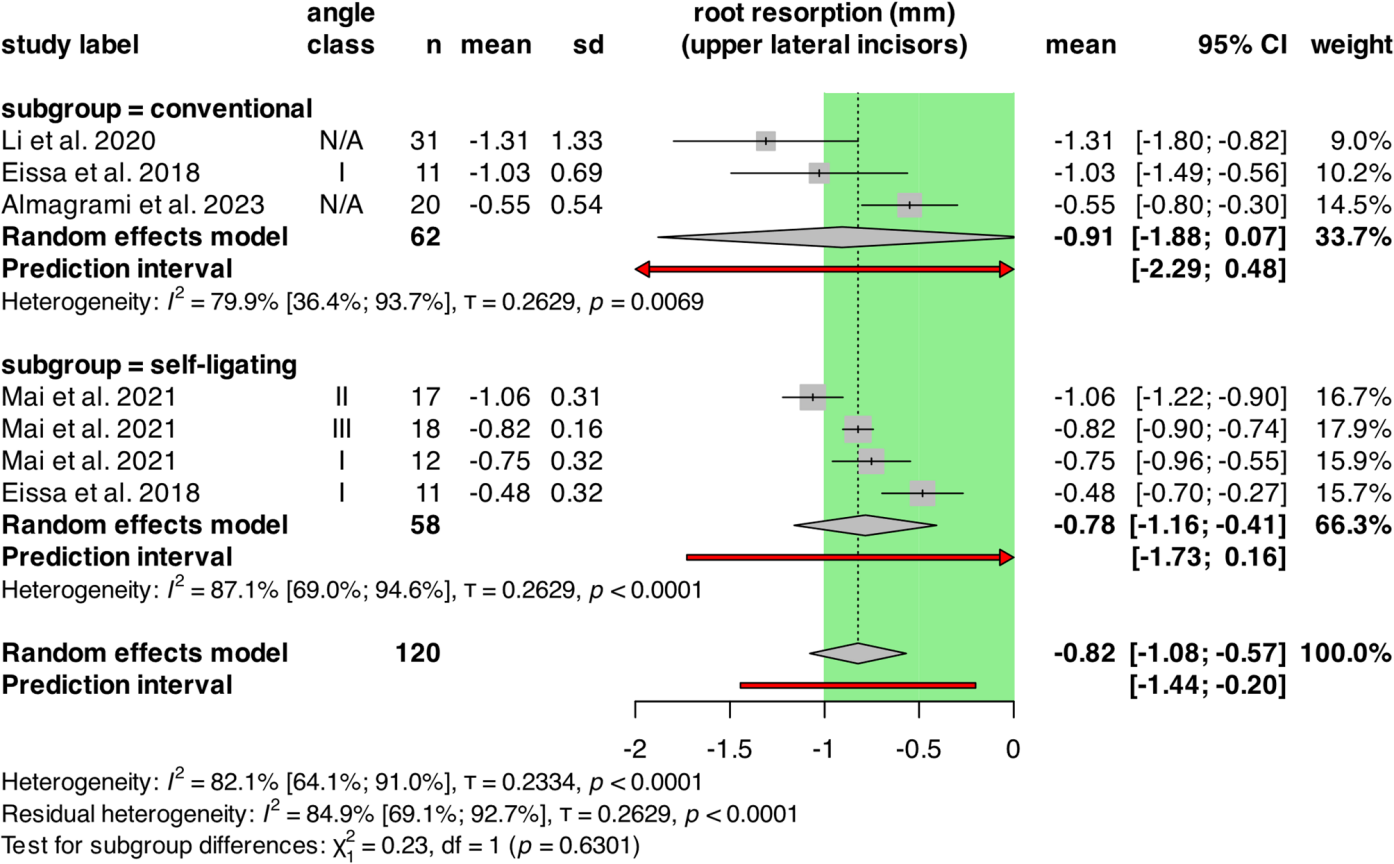
Forest plot showing OIIRR of upper lateral incisors in conventional versus self-ligating groups. The green area represents the cut-off value of clinically not relevant extent of root resorption. CI: confidence intervals; n: number of patients; SD: standard deviation

The subgroup analysis of CFA and SL groups is shown in Fig. 5. The analysis included 120 patients from 4 retrospective cohort studies. The CFA group had an I^2^ of 79.9% [CI: 36.4%; 93.7%; τ = 0.2629, *p* = 0.0069] and a mean resorption of -0.91 mm [CI: -1.88; 0.07], the SL had an I^2^ of 87.1% [CI: 69.0%; 94.6%; τ = 0.2629, *p* < 0.0001] and a mean resorption of -0.78 mm [CI: -1.16; -0.41].

There was no statistically significant difference between the groups (*p* = 0.6301).

The random effects model resulted in -0.82 mm [CI:-1.08; -0.57] overall mean resorption. Overall heterogeneity was significant, with I^2^ = 82.1% [CI: 64.1%; 91.0%, τ = 0.2334, *p* < 0.0001].

The multilevel/multivariate model showed similar effects: mean resorption in the CFA group was − 1.00 mm, while − 0.62 mm in the SL group and the difference was not significant (*p* = 0.1258).

For the pairwise analysis, only one study was available with 22 patients, so no pooling was possible. (Supplementary Fig. 4.)

### Root resorption of other tooth types

Two articles measured both upper and lower front teeth besides the upper incisors. For aligners, Li Yuan et al. [7] found statistically significant root resorption only in the case of upper central incisors (0.26 mm ± 0.42 mm), upper lateral incisors (0.23 mm ± 0.37 mm) and lower central incisors (0.20 mm ± 0.45 mm). In contrast, all six tooth positions showed significant levels of OIIRR in the conventional fixed appliance (CFA) group, with the highest value of 1.53 mm ± 1.92 mm, measured on upper canines. On the basis of prevalence data, there were severe cases only in the CFA group (Sharpe’s 3rd degree: 0.81%).

Mai et al. [31] observed root resorption in all six front tooth positions without a significant difference between the treatment groups (*P* > 0.05). Although aligners caused less OIIRR in Angle Class I patients than SL, upper anterior teeth of Class II and upper and lower anterior teeth of Class III patients presented more root resorption in the aligner groups than with self-ligating appliances. The highest amount of root resorption was present in the case of upper central incisors (1.05 mm ± 0.27 mm) in the aligner group and upper lateral incisors (1.05 mm ± 0.29 mm) in the self-ligating group.

### Root resorption on radiographs

The RCT by Toyokawa et al. [8] used periapical radiographs six months after treatment initiation to investigate the OIRR of upper and lower incisors using conventional fixed appliances and aligners. The results suggest a similarly low degree of OIRR for both groups with a clinically insignificant reduction of root length. The overall differences between groups were not clinically relevant either, ranging from 0.03 to 0.35 mm.

### Root resorption as a volumetric measurement

Chen et al. [16] used further volumetric measurements to assess changes in upper central incisors. A significant reduction in volume and length was present in the aligner, CFA, and SL groups. Although length changes did not differ significantly between the groups, root resorption was significantly lower volumetrically in the aligner group.

### Risk of bias assessment

One article presented a low overall risk, four a moderate risk, and one a high risk of bias due to the possible selection of results reported. The analyzed studies were retrospective, and the bias due to the “selection of participants” and “selection of reported results” domains were at least moderate in all of them, based on the risk of bias assessment. The reported results may be subject to selection and publication biases. Detailed results of the RoB assessment are shown in Supplementary Fig. 5.

### Publication bias and certainty of evidence

Egger’s test was not applicable due to the low number of studies.

Grading of Recommendations, Assessment, Development and Evaluation (GRADE) assessment was not applicable, as there was no direct comparison between the interventions studied.

## Discussion

Previous studies emphasized that the use of aligners may reduce the risk of OIIRR [7, 16, 17]. However, our study found no statistically significant or clinically relevant difference in OIIRR of upper central and lateral incisors between fixed appliances and aligners. Despite the use of different cut-off values to determine clinical significance [18, 32], resorption was not clinically relevant in any of the groups studied, even with a rigorous 1 mm cut-off value.

The results, however, may be affected by heterogeneity, which can be explained by the low number of studies included, variables in patient demographics, duration of treatment, severity of initial malocclusions and differences in patient exclusion criteria. (Supplementary Tables 4–5) Confounding factors, such as differing delivered forces, archwire sequences, aligner staging protocols, auxiliaries (for example, intermaxillary elastics), and tooth movements, were not standardized within and between the included studies.

The absence of clinically relevant differences between conventional and self-ligating fixed appliances, which has been conducted previously [9], also suggests that treatment modalities should be chosen based on necessary tooth movements, the experience of the orthodontist, compliance of the patient and other indications, rather than the expectations regarding root resorption.

The efficacy of the treatment options, however, may not be equal. Studies suggesting similar outcomes with aligners and fixed appliances [33, 34] are constantly challenged [35]. The increasing predictability due to improved aligner generations is still low, depending on the desired movement. The overall accuracy of movements can be esteemed at 50% [36]. 

Newer, innovative attachment designs can change movement efficacy, although planned overcorrections and refinements often cannot be avoided while using aligners [37]. 

The amount of aligner treatments switched for fixed appliances should not be neglected either. Kravitz et al. reported that one in every six of their patients continues their aligner treatment by switching to braces [38], as even multiple refinements may not deliver the expected outcomes. The clinical question of root resorption differences between aligners and braces may be opposed, because planned aligner movements (especially root movements) often do not occur accurately [39, 40], as they would with fixed braces. Regardless, carefully planned anchorage and sequencing of movements (such as intrusion) may help maintain low delivered forces and decrease the triggers of OIIRR.

The continuous development of aligner materials also creates substantial differences in biomechanics between these systems. The latest advancement in aligner orthodontics is direct 3D-printed aligners that may revolutionize treatment options [41–43], as deformation of the conventional aligner materials is one of the main obstacles that hinders predictability [44], and rapid force decay can be observed with non-shape-memory aligners [45]. Individualized trimming lines and the control of aligner thickness may also be beneficial in terms of predictability [46, 47]. No previous studies have investigated root resorption with 3D-printed shape memory aligners. Thus, possible differences are yet to be discovered.

Biomechanics, should be carefully planned during the application of any appliance, as heavy forces, extraction treatments, intrusion or torque movements, and movements that cause long apical displacements increase the risk of OIIRR and should be avoided if root resorption is expected [48]. 

Different populations may show great differences in root resorption due to risk factors. As most of the studies enrolled patients who did not present well-known risk factors, such as pre-existing root resorption, dental trauma, previous orthodontic treatment, missing teeth, and severe crowding or other confounding factors, such as endodontic treatments and developmental anomalies, the results should be carefully implemented in the treatment of risk populations. (see Supplementary Table 5) Until future research helps us to determine the differences within risk groups, risk factors should be identified to assess the individual risk profile of each patient so biomechanics, regardless of the used system, can be planned accordingly. Early detection of OIIRR is also important, as evidence suggests that pausing orthodontic forces upon recognition of root resorption may stop further hard tissue loss, with limited possibilities of regeneration [49]. 

Although the results did not reach statistical significance, the trend in root length changes suggests that differences (Figs. 2 and 4) may reach a level of clinical relevance in populations predisposed to OIIRR. In this case, the use of aligners over fixed appliances may be beneficial. However, further studies in patients with the aforementioned risk factors should be conducted in a reliably randomized, prospective manner to support this assumption.

The differences in our study may be explained by the fact that aligners tend to be less effective in root movements [36], to an extent that the forces triggering root resorption may be minor. The possibility that reporting bias has distorted the results, suggesting the superiority of aligners, cannot be rejected either.

As OIIRR is severe (exceeds 4 mm) in only 1–5% of cases [9], there is a low number of clinically relevant root resorptions within the normal orthodontic population; thus, studies investigating prevalence, rather than root length change as an outcome, may provide more valuable information to better understand this adverse effect. Classification systems for external apical root resorption, such as the one introduced by Sharpe et al. [50], are often used to assess severity and may also be valuable tools for future research. Only two studies provided data on prevalence [7, 30], both using the method by Sharpe. Interestingly, in both articles, only conventional fixed appliances showed grade 3 (severe) root resorptions, meaning that more than ¼ of the root was resorbed.

Although there was no apparent difference between the articles included, standardizing root resorption measurements between studies may also be beneficial, as using reference points of the clinical crown over the cementoenamel junction may theoretically distort the results due to enamel loss during the treatment.

As root resorption does not only cause longitudinal changes, volumetric measurements may provide a more accurate assessment of tissue loss [51]. Hongyu et al. found statistically significant volume loss in UCI roots even in patients who did not present a significant reduction of root length [16]. On the other hand, Khalil et al. found no significant difference between aligners and fixed appliances within an RCT using volumetric measurements [52]. Recent results of Lin et al. suggest a statistically significant volumetric decrease of the roots in the orthodontically treated population, introducing a deep learning-based tooth segmentation and volumetric analysis [53]. Volumetric measurements with surface-based superimposition may overcome the difficulties of landmark identification and result in higher reproducibility of measurements. Thus, these measurements can be superior to 2D and 3D length measurements [54]. 

Although volumetric measurements may change the direction of root resorption-related research, clinically insignificant results do not support the routine use of CBCT measurements for OIIRR control without any additional indications yet [55], as the ALARA (As Low As Reasonably Achievable) guidance should be followed in orthodontic diagnostics [56].

Efforts have been made to create supplementary interventions to prevent or reverse OIIRR, for example, the use of Low-Intensity Pulsed Ultrasound (LIPUS) is emerging, and so is the evidence supporting its role in clinical practice [57]. On the other hand, as in most cases, OIIRR rarely reaches a level of clinical significance in the general population. Thus, the cost-benefit of these supplements for patients should be evaluated on an individual basis.

### Strengths and limitations

The strengths of our study were that we only needed to deviate slightly (see Methods) from our prospective protocol and used a rigorous methodology. We are not aware of any other purely CBCT-based analysis that used only dual-arm studies and incorporated the latest findings. Subgroup analysis of fixed appliances provided additional valuable information on the performance of conventional and self-ligating fixed appliances.

Considering the limitations of this work, we have to take into account the small body and high heterogeneity of the available evidence processed, as well as the retrospective nature and moderate to high risk of bias of the articles included. Individual differences may confound the results, as neither baseline malocclusions, demographics, nor supplementary interventions (e.g., extractions, intermaxillary elastics) were standardized between the studies.

### Implications for practice and research

Translating scientific findings into clinical practice is crucial [58, 59]. On the basis of our results, we suggest that within the risk-free population, clinicians should choose the treatment method based on the expected outcome and their expertise and not prioritize aligners or fixed appliances over the other to reduce the probability of root resorption. However, the trend in the results presented suggests that aligners may be beneficial for patients at risk of OIIRR if the severity of the discrepancy and the expertise of the orthodontist allow this type of treatment. We do not advocate the use of self-ligating appliances over conventional fixed appliances either, solely to reduce the risk of OIIRR. CBCT screenings for OIIRR are not justified without other indications.

Better standardization of populations, interventions, and outcome measurements is needed for future research. Although randomization of such treatments is challenging, high-quality, prospective studies are needed to reduce the probability of reporting bias. Studies should also provide prevalence data, as it may be a more sensitive metric in this case.

## Conclusions

The data suggest that there is no clinically significant difference between fixed appliances and aligners in OIIRR. Conventional and self-ligating fixed appliances showed no significant difference in OIIRR. All treatment modalities caused similar, clinically irrelevant levels of OIIRR in the investigated population. The treatment modality should be selected based on biomechanics, expected outcomes, and individual preferences.

The results should be interpreted cautiously due to the risk of bias and heterogeneity.

## Electronic supplementary material

Below is the link to the electronic supplementary material.

Supplementary Material 1

## Data Availability

The datasets used in this study can be found in the full-text articles included in the systematic review and meta-analysis.

## Abbreviations

ALARA: As Low As Reasonably Achievable
C: Control group
CBCT: Cone beam computed tomography
CFA: Conventional fixed appliances
CI: Confidence intervals
GRADE: Grading of Recommendations, Assessment, Development and Evaluation
HK: Hartung-Knapp adjustment
I: Intervention group
IO: Intraoral
LIPUS: Low-Intensity Pulsed Ultrasound
MRAW: Raw mean
NA: Not available
OIIRR: Orthodontically induced inflammatory root resorption
PRISMA: Preferred Reporting Items for Systematic reviews and Meta-Analyses
RCT: Randomized controlled trials
RoB2: Revised Cochrane Risk Of Bias Tool For Randomized Trials
ROBINS-I: The Risk Of Bias In Non-randomized Studies - of Interventions
SE: Standard error
SL: Self-ligating appliances
UCI: Upper central incisors
ULI: Upper lateral incisors
