# Supplementary material for: Root resorption caused by aligners, self-ligating appliances, and conventional fixed appliances: a CBCT-based meta-analysis
Source: BMC Oral Health. 2025 Jul 26;25:1259. doi: 10.1186/s12903-025-06639-2 (PMC12296614; doi:10.1186/s12903-025-06639-2)
Supplement: Supplementary file 1 — Supplementary Material 1 [file 12903_2025_6639_MOESM1_ESM.docx]

**SUPPLEMENTARY MATERIAL**

**Supplementary Table 1.** PRISMA checklist

| **Section and Topic** | **Item #** | **Checklist item** | **Location where item is reported** |
| --- | --- | --- | --- |
| **TITLE** | | |  |
| Title | 1 | Identify the report as a systematic review. | Page 1 |
| **ABSTRACT** | | |  |
| Abstract | 2 | See the PRISMA 2020 for Abstracts checklist. | Page 1-2 |
| **INTRODUCTION** | | |  |
| Rationale | 3 | Describe the rationale for the review in the context of existing knowledge. | Page 3-4 |
| Objectives | 4 | Provide an explicit statement of the objective(s) or question(s) the review addresses. | Page 3-4 |
| **METHODS** | | |  |
| Eligibility criteria | 5 | Specify the inclusion and exclusion criteria for the review and how studies were grouped for the syntheses. | Page 4-5 |
| Information sources | 6 | Specify all databases, registers, websites, organisations, reference lists and other sources searched or consulted to identify studies. Specify the date when each source was last searched or consulted. | Page 5 |
| Search strategy | 7 | Present the full search strategies for all databases, registers and websites, including any filters and limits used. | Page 5 |
| Selection process | 8 | Specify the methods used to decide whether a study met the inclusion criteria of the review, including how many reviewers screened each record and each report retrieved, whether they worked independently, and if applicable, details of automation tools used in the process. | Page 5-6 |
| Data collection process | 9 | Specify the methods used to collect data from reports, including how many reviewers collected data from each report, whether they worked independently, any processes for obtaining or confirming data from study investigators, and if applicable, details of automation tools used in the process. | Page 6 |
| Data items | 10a | List and define all outcomes for which data were sought. Specify whether all results that were compatible with each outcome domain in each study were sought (e.g. for all measures, time points, analyses), and if not, the methods used to decide which results to collect. | Page 6 |
|  | 10b | List and define all other variables for which data were sought (e.g. participant and intervention characteristics, funding sources). Describe any assumptions made about any missing or unclear information. | Page 6 |
| Study risk of bias assessment | 11 | Specify the methods used to assess risk of bias in the included studies, including details of the tool(s) used, how many reviewers assessed each study and whether they worked independently, and if applicable, details of automation tools used in the process. | Page 6 |
| Effect measures | 12 | Specify for each outcome the effect measure(s) (e.g. risk ratio, mean difference) used in the synthesis or presentation of results. | Page 6-7 |
| Synthesis methods | 13a | Describe the processes used to decide which studies were eligible for each synthesis (e.g. tabulating the study intervention characteristics and comparing against the planned groups for each synthesis (item #5)). | Page 6-7 |
|  | 13b | Describe any methods required to prepare the data for presentation or synthesis, such as handling of missing summary statistics, or data conversions. | Page 6-7 |
|  | 13c | Describe any methods used to tabulate or visually display results of individual studies and syntheses. | Page 6-7 |
|  | 13d | Describe any methods used to synthesize results and provide a rationale for the choice(s). If meta-analysis was performed, describe the model(s), method(s) to identify the presence and extent of statistical heterogeneity, and software package(s) used. | Page 6-7 |
|  | 13e | Describe any methods used to explore possible causes of heterogeneity among study results (e.g. subgroup analysis, meta-regression). | Page 6-7 |
|  | 13f | Describe any sensitivity analyses conducted to assess robustness of the synthesized results. | Page 6-7 |
| Reporting bias assessment | 14 | Describe any methods used to assess risk of bias due to missing results in a synthesis (arising from reporting biases). | Page 6-7 |
| Certainty assessment | 15 | Describe any methods used to assess certainty (or confidence) in the body of evidence for an outcome. | Not applicable |
| **RESULTS** | | |  |
| Study selection | 16a | Describe the results of the search and selection process, from the number of records identified in the search to the number of studies included in the review, ideally using a flow diagram. | Page 8 |
|  | 16b | Cite studies that might appear to meet the inclusion criteria, but which were excluded, and explain why they were excluded. | Page 8 |
| Study characteristics | 17 | Cite each included study and present its characteristics. | Page 8 |
| Risk of bias in studies | 18 | Present assessments of risk of bias for each included study. | Suppl. fig. 5. |
| Results of individual studies | 19 | For all outcomes, present, for each study: (a) summary statistics for each group (where appropriate) and (b) an effect estimate and its precision (e.g. confidence/credible interval), ideally using structured tables or plots. | Page 8-13 |
| Results of syntheses | 20a | For each synthesis, briefly summarise the characteristics and risk of bias among contributing studies. | Page 8-13 |
|  | 20b | Present results of all statistical syntheses conducted. If meta-analysis was done, present for each the summary estimate and its precision (e.g. confidence/credible interval) and measures of statistical heterogeneity. If comparing groups, describe the direction of the effect. | Page 8-13 |
|  | 20c | Present results of all investigations of possible causes of heterogeneity among study results. | Page 8-13 |
|  | 20d | Present results of all sensitivity analyses conducted to assess the robustness of the synthesized results. | Page 8-13 |
| Reporting biases | 21 | Present assessments of risk of bias due to missing results (arising from reporting biases) for each synthesis assessed. | Page 13 |
| Certainty of evidence | 22 | Present assessments of certainty (or confidence) in the body of evidence for each outcome assessed. | Not applicable |
| **DISCUSSION** | | |  |
| Discussion | 23a | Provide a general interpretation of the results in the context of other evidence. | Page 14-18 |
|  | 23b | Discuss any limitations of the evidence included in the review. | Page 18 |
|  | 23c | Discuss any limitations of the review processes used. | Page 18 |
|  | 23d | Discuss implications of the results for practice, policy, and future research. | Page 18-19 |
| **OTHER INFORMATION** | | |  |
| Registration and protocol | 24a | Provide registration information for the review, including register name and registration number, or state that the review was not registered. | Page 2 |
|  | 24b | Indicate where the review protocol can be accessed, or state that a protocol was not prepared. | Page 4 |
|  | 24c | Describe and explain any amendments to information provided at registration or in the protocol. | Page 4 |
| Support | 25 | Describe sources of financial or non-financial support for the review, and the role of the funders or sponsors in the review. | Not applicable |
| Competing interests | 26 | Declare any competing interests of review authors. | None to declare |
| Availability of data, code and other materials | 27 | Report which of the following are publicly available and where they can be found: template data collection forms; data extracted from included studies; data used for all analyses; analytic code; any other materials used in the review. | Page 6 |

**Supplementary Document 1.** Search keys used for systematic search

PubMed: ("aligner*" OR "invisible" OR "transparent" OR "removable" OR "AngelAlign" OR "ClearPath" OR "SmartTrack" OR "Invisalign" OR "ClearCorrect" OR "SureSmile" OR "Orthocaps") AND (("fixed" AND "appliance*") OR "multiband" OR "multibond" OR "brace*" OR "bracket*" OR "archwire*" OR "Roth" OR "Alexander" OR "Damon" OR "selfligating" OR "edgewise" OR "tip-edge" OR "straight-wire" OR "straightwire")

Embase (mapping turned off): (aligner* OR invisible OR transparent OR removable OR angelalign OR clearpath OR smarttrack OR invisalign OR clearcorrect OR suresmile OR orthocaps) AND (multiband OR multibond OR brace* OR bracket* OR archwire* OR selfligating OR edgewise OR tip-edge OR straight-wire OR straightwire OR roth OR alexander OR damon OR (fixed AND appliance*))

Cochrane: ('aligner*' OR 'invisible' OR 'transparent' OR 'removable' OR 'angelalign' OR 'clearpath' OR 'smarttrack' OR 'invisalign' OR 'clearcorrect' OR 'suresmile' OR ’orthocaps’) AND ('fixed' AND 'appliance*' OR 'multiband' OR 'multibond' OR 'brace*' OR 'bracket*' OR 'archwire*' OR 'selfligating' OR 'edgewise' OR 'tip-edge' OR 'straight-wire' OR 'straightwire' OR 'roth' OR 'alexander' OR 'damon')

Scopus: (aligner* OR invisible OR transparent OR removable OR angelalign OR clearpath OR smarttrack OR invisalign OR clearcorrect OR suresmile OR orthocaps) AND (multiband OR multibond OR brace* OR bracket* OR archwire* OR selfligating OR edgewise OR tip-edge

**Supplementary Table 2.** Reasons for full-text exclusions

| First author (year) | DOI/PMID | Title | Reason for exclusion |
| --- | --- | --- | --- |
| Apajalahti et al. (2007) | 10.1093/ejo/cjm016 | Apical root resorption after orthodontic treatment-a retrospective study | Incorrect population |
| Costello et al. (2020) | 10.21307/aoj-2020-014 | The incidence and severity of root resorption following orthodontic treatment using clear aligners | Single arm |
| Currier et al. (2013) | 22567621 | Anterior and posterior root resorption with cone beam computed tomography | Incorrect intervention |
| Faxén et al. (2018) | 10.1093/ejo/cjx086 | Incisor root resorption in class II division 2 patients in relation to orthodontic treatment | Single arm |
| Guo et al. (2023) | 10.1111/ocr.12705 | A retrospective study of alveolar bone remodelling after anterior retraction in orthodontic tooth extraction cases with clear aligners and fixed appliances | Incorrect outcome |
| Gupta et al. (2023) | 10.6026/97320630019764 | 3D assessment of alveolar bone alterations in orthodontic movement among Indians | Incorrect outcome |
| Iglesias-Linares et al. (2017) | 10.2319/02016-101.1 | Orthodontically induced external apical root resorption in patients treated with fixed appliances vs removable aligners | Incorrect outcome |
| Khalil et al. (2023) | 10.47750/pnr.2023.14.S02.92 | Apical Root Resorption Accompanied Orthodontic Treatment Using Clear Aligners Versus Fixed Appliances: A Cbct Comparative Study | Incorrect outcome |
| Linge et al. (1983) | 10.1093/ejo/5.3.173 | Apical root resorption in upper anterior teeth | Incorrect population |
| Li et al. (2020) | 10.19439/j.sjos.2020.06.018 | Effect of different orthodontic techniques on alveolar bone changes in the upper incisor area of patients with periodontitis | Incorrect outcome |
| Makedonas et al. (2009) | 20162928 | Management of root resorption in a large orthodontic clinic | Incorrect outcome |
| Ohm et al. (1980) | 10.1093/ejo/5.3.173 | Apical root resorption of the upper incisors | Single arm |
| Patel et al. (2012) | 22567621 | A CBCT comparison of anterior root resorption in SureSmile and conventional edgewise treatments | Incorrect intervention |
| Preoteasa et al. (2009) | 19434320 | Orthodontically induced root resorption correlated with morphological characteristics | Incorrect intervention |
| Smeyers et al. (2022) | 10.1007/s00784-022-04679-4. | Evolution of root length throughout orthodontic treatment in maxillary incisors with previous history of dental trauma: a longitudinal controlled trial | Incorrect intervention |
| Wang et al. (2017) | 28474083 | A retrospective study on incisor root resorption in patients treated with bracketless invisible appliance and straight wire appliance | Incorrect outcome |
| Withayanukonkij et al. (2023) | 10.2319/010723-14.1 | Root resorption during maxillary molar intrusion with clear aligners: a randomized controlled trial | Incorrect intervention |
| Alfawal et al. (ongoing trial) | NA | A comparison between clear aligners and conventional fixed appliances - a randomized controlled trial | Unavailable results |
| Upadhyay et al. (ongoing trial) | NA | Comparison of Oral Hygiene & Root Resorption During Orthodontic Treatment | Unavailable results |
| Obaida et al. (ongoing trial) | NA | Root Resorption Accompanied Clear Aligners and Fixed Orthodontic Appliance | Unavailable results |
| Abdelmotaleb et al. (ongoing trial) | NA | Effectiveness of Low Frequency Vibration on the Rate of Canine Retraction | Incorrect outcome |
| Pedron et al. (ongoing trial) | NA | Effects of orthodontic treatment with clear aligner and conventional fixed appliance | Unavailable results |
| Jyotirmay et al. (2021) | 34615781 | Comparison of Apical Root Resorption in Patients Treated with Fixed Orthodontic Appliance and Clear Aligners: A Cone-beam Computed Tomography Study | Suspected plagiarism |

**Supplementary Table 3.** Raw pretreatment root and tooth length data. CFA: conventional fixed appliance; SD: standard deviation; SL: self-ligating; UCI: upper central incisor; ULI: upper lateral incisor

|  | UCI tooth length before treatment (mm) | | | | | |
| --- | --- | --- | --- | --- | --- | --- |
|  | **Aligner** | | **CFA** | | **SL** | |
|  | **Mean** | **SD** | **Mean** | **SD** | **Mean** | **SD** |
| Chen, H., et al. | 22.06 | 0.80 | 22.17 | 1.01 | 21.85 | 0.87 |
| Eissa, O., et al. (right side) | 23.35 | 1.79 | 25.06 | 3.17 | 25.00 | 2.13 |
| Eissa, O., et al. (left side) | 23.46 | 1.85 | 24.76 | 2.96 | 24.84 | 2.30 |
| Li, Y., et al. | 21.55 | 1.86 | 21.25 | 1.85 | NA | NA |
| Toyokawa-S., K.C., et al. (right side) | 26.39 | 2.24 | 24.72 | 2.58 | NA | NA |
| Toyokawa-S., K.C., et al. (left side) | 26.44 | 2.42 | 25.28 | 2.27 | NA | NA |
|  | ULI tooth length before treatment (mm) | | | | | |
|  | **Aligner** | | **CFA** | | **SL** | |
|  | **Mean** | **SD** | **Mean** | **SD** | **Mean** | **SD** |
| Chen, H., et al. | NA | NA | NA | NA | NA | NA |
| Eissa, O., et al. (right side) | 21.60 | 1.28 | 22.45 | 2.57 | 22.75 | 1.69 |
| Eissa, O., et al. (left side) | 21.98 | 1.50 | 22.72 | 2.70 | 22.77 | 1.73 |
| Li, Y., et al. | 20.60 | 1.94 | 20.23 | 1.53 | NA | NA |
| Toyokawa-S., K.C., et al. (right side) | 25.38 | 2.20 | 24.19 | 2.40 | NA | NA |
| Toyokawa-S., K.C., et al. (left side) | 25.74 | 1.75 | 24.61 | 2.36 | NA | NA |
|  |  |  |  |  |  |  |
|  | UCI root length before treatment (mm) | | | | | |
|  | **Aligner** | | **CFA** | | **SL** | |
|  | **Mean** | **SD** | **Mean** | **SD** | **Mean** | **SD** |
| Almagrami, I., et al. | 12.14 | 1.67 | 11.82 | 1.68 | NA | NA |
| Mai, T., et al. | 11.21 | 0.33 | NA | NA | 11.27 | 1.49 |
|  | ULI root length before treatment (mm) | | | | | |
|  | **Aligner** | | **CFA** | | **SL** | |
|  | **Mean** | **SD** | **Mean** | **SD** | **Mean** | **SD** |
| Almagrami, I., et al. | 12.72 | 1.24 | 12.50 | 1.36 | NA | NA |
| Mai, T., et al. | 11.57 | 1.49 | NA | NA | 11.44 | 1.51 |

**Supplementary Figure 1.** Forest plot showing OIIRR of upper central incisors in aligners versus fixed appliances. Effect size measure: mean difference. The green area represents the cut-off value of clinically not relevant difference in root resorption. SL: self-ligating; CFA: conventional fixed appliance; CI: confidence intervals; n: number of patients; SD: standard deviation; MD: mean difference


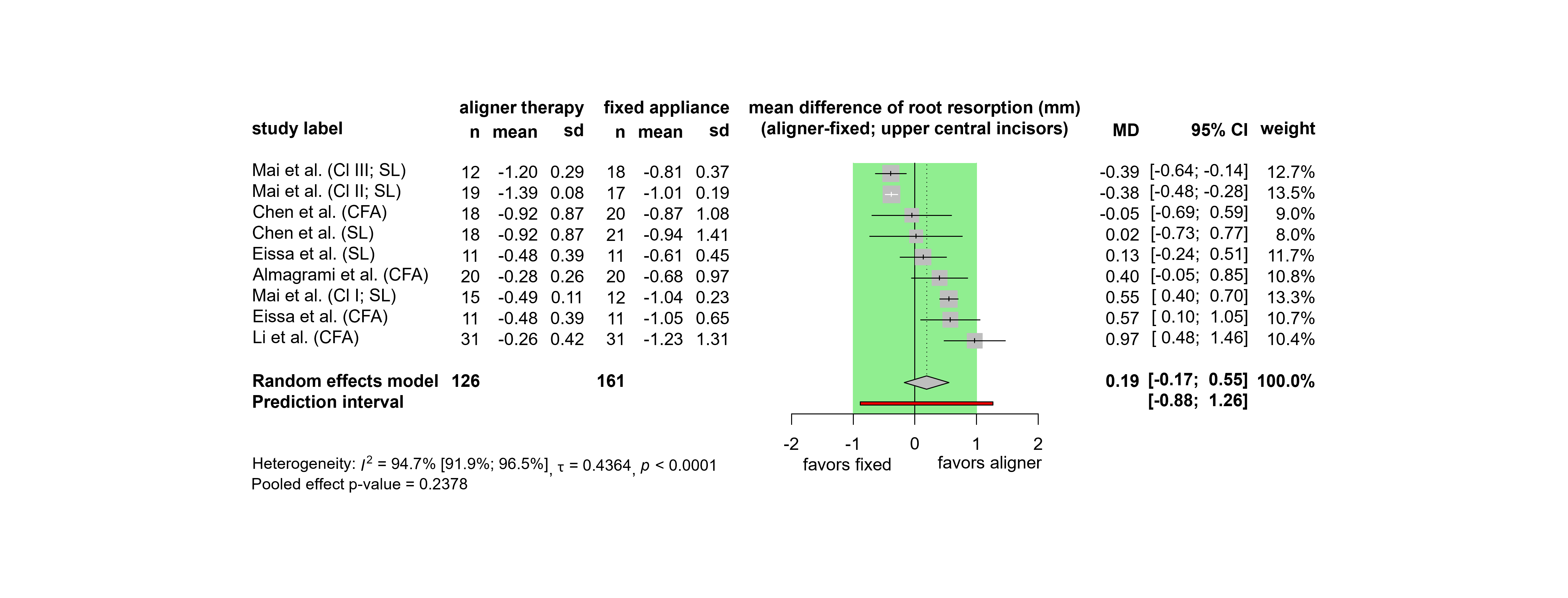


**Supplementary Figure 2.** Forest plot showing OIIRR of upper central incisors in self-ligating versus conventional groups. Effect size measure: mean difference. The green area represents the cut-off value of clinically not relevant difference in root resorption. SL: self-ligating; CFA: conventional fixed appliance; CI: confidence intervals; n: number of patients; SD: standard deviation; MD: mean difference


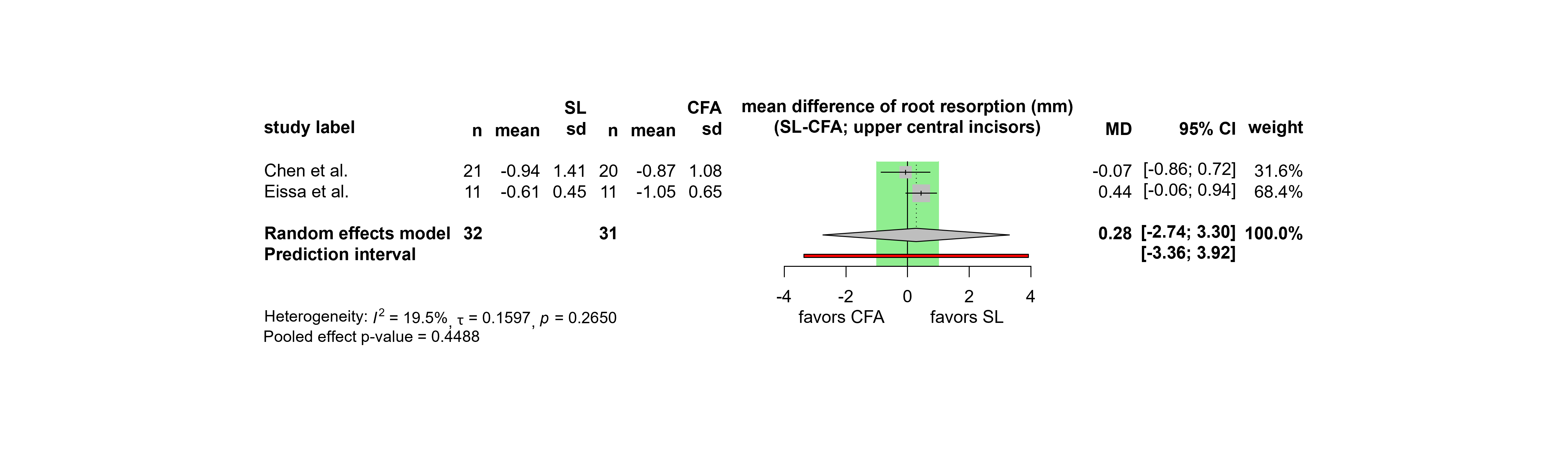


**Supplementary Figure 3.** Forest plot showing OIIRR of upper lateral incisors in aligners versus fixed appliances. Effect size measure: mean difference. The green area represents the cut-off value of clinically not relevant difference in root resorption. SL: self-ligating; CFA: conventional fixed appliance; CI: confidence intervals; n: number of patients; SD: standard deviation; MD: mean difference


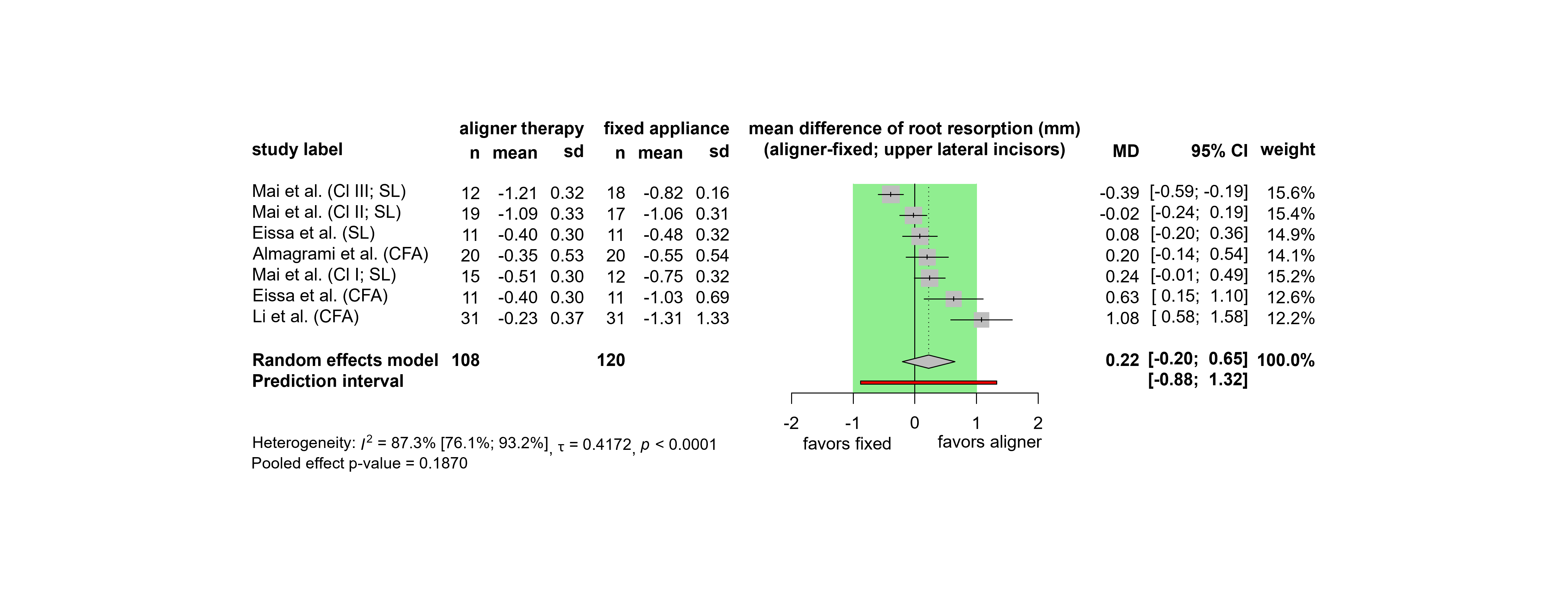


**Supplementary Figure 4.** Forest plot showing OIIRR of upper lateral incisors in self-ligating versus conventional groups. Effect size measure: mean difference. The green area represents the cut-off value of clinically not relevant difference in root resorption. SL: self-ligating; CFA: conventional fixed appliance; CI: confidence intervals; n: number of patients; SD: standard deviation; MD: mean difference


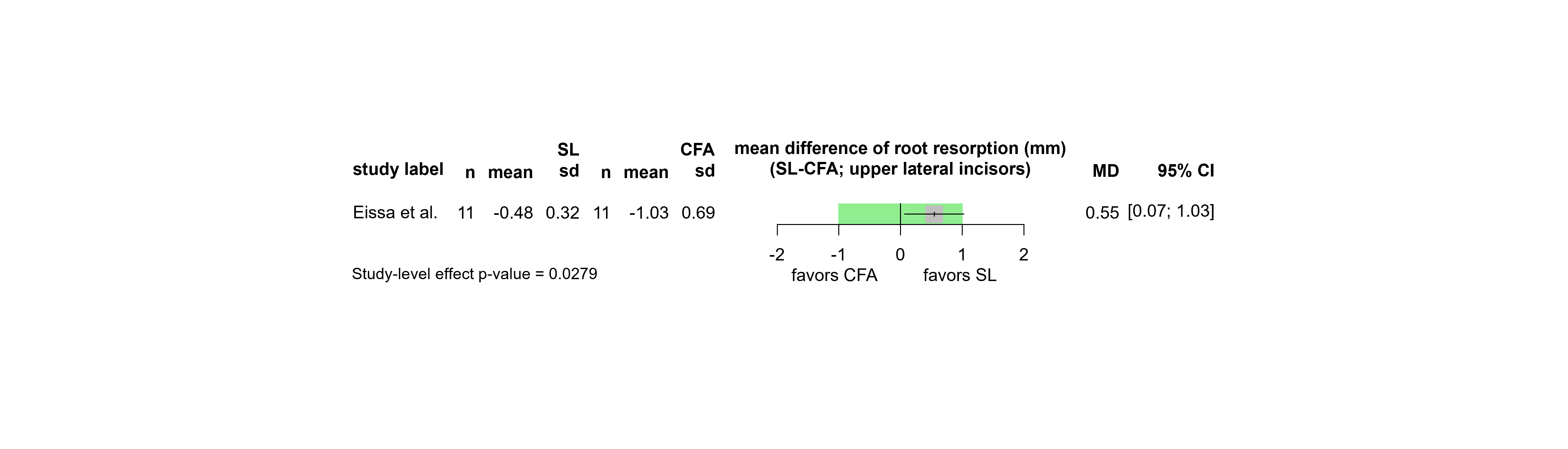


**Supplementary Figure 5.** Risk of bias assessment. D: domain


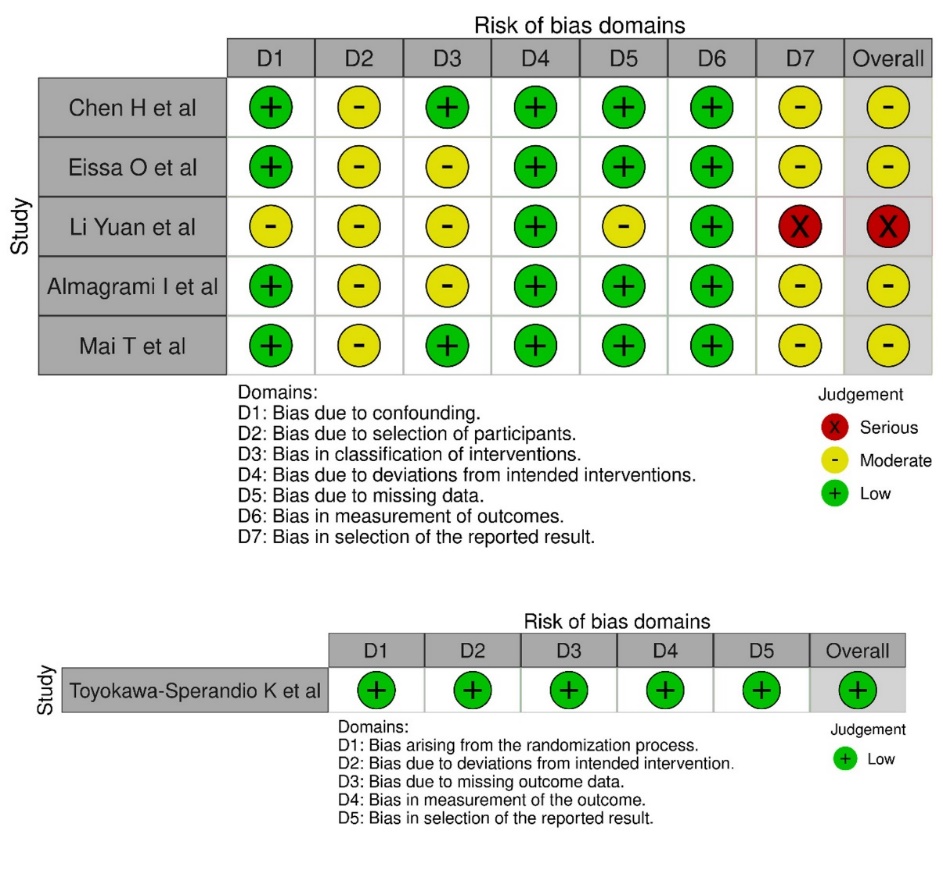


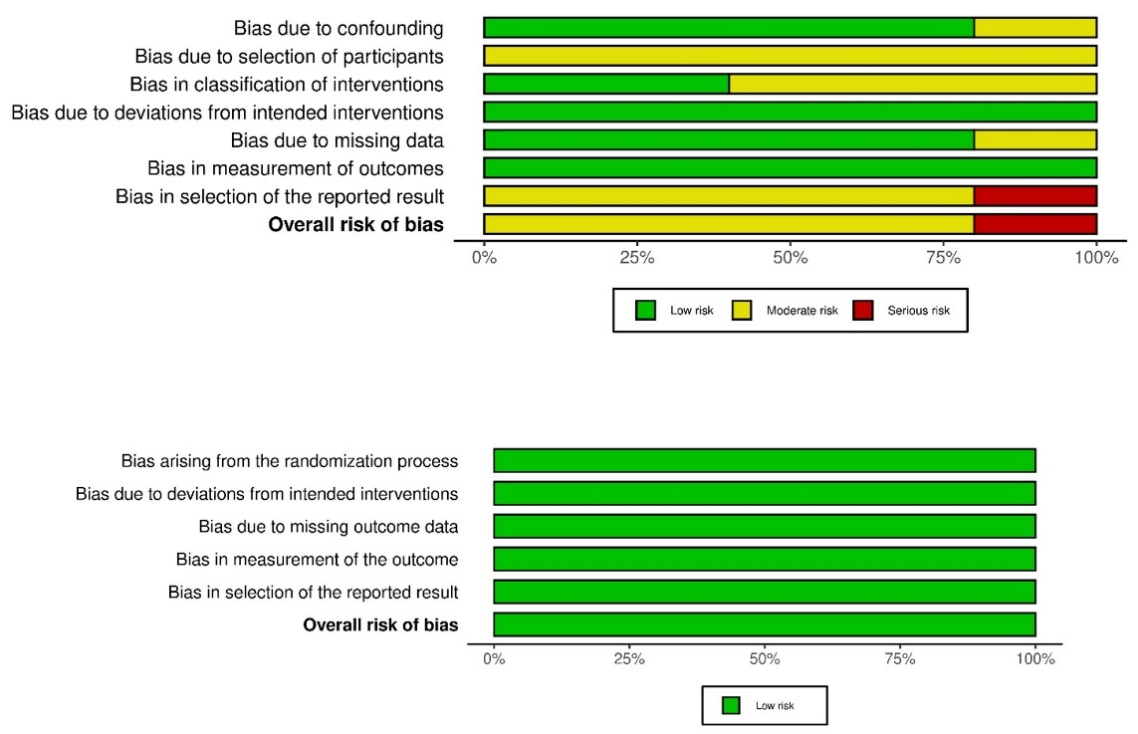


**Supplementary Table 4.** Possible confounding factors. ABO DI: American Board of Orthodontics Discrepancy Index; CFA: conventional fixed appliance; SD: standard deviation; SL: self-ligating

|  | Treatment duration in months | | | | | |
| --- | --- | --- | --- | --- | --- | --- |
|  | **Aligner** | | **CFA** | | **SL** | |
|  | **Mean** | **SD** | **Mean** | **SD** | **Mean** | **SD** |
| Chen, H., et al. | 30.9 | 4.3 | 28.3 | 5.1 | 27.7 | 4.7 |
| Eissa, O., et al. | 15.1 | 1.9 | 16.2 | 2.8 | 15.8 | 1.7 |
| Li, Y., et al. | 21.5 | 5.6 | 23.3 | 6.3 | NA | NA |
| Almagrami, I., et al. | 25.9 | 8.0 | 29.7 | 7.7 | NA | NA |
| Mai, T., et al. | 22.2 | 4.4 | NA | NA | 22.9 | 4.8 |
| Toyokawa-S., K.C., et al. | 6.0 | 0.0 | 6.0 | 0.0 | NA | NA |
|  | Age in years | | | | | |
|  | **Aligner** | | **CFA** | | **SL** | |
|  | **Mean** | **SD** | **Mean** | **SD** | **Mean** | **SD** |
| Chen, H., et al. | 22.7 | 3.1 | 23.6 | 3.2 | 23.7 | 3.4 |
| Eissa, O., et al. | 18.3 | 2.8 | 17.3 | 2.4 | 17.7 | 2.2 |
| Li, Y., et al. | 24.7 | 7.5 | 22.5 | 6.5 | NA | NA |
| Almagrami, I., et al. | 25.2 | 6.7 | 22.3 | 4.3 | NA | NA |
| Mai, T., et al. | 26.8 | 5.8 | NA | NA | 28.5 | 6.4 |
| Toyokawa-S., K.C., et al. | 23.6 | 5.7 | 20.6 | 4.5 | NA | NA |
|  | ABO DI score | | | | | |
|  | **Aligner** | | **CFA** | | **SL** | |
|  | **Mean** | **SD** | **Mean** | **SD** | **Mean** | **SD** |
| Chen, H., et al. | 12.0 | 3.4 | 13.7 | 3.0 | 13.5 | 3.1 |
| Eissa, O., et al. | - | - | - | - | - | - |
| Li, Y., et al. | 18.8 | 7.7 | 17.1 | 10.1 | - | - |
| Almagrami, I., et al. | 12.7 | 0.5 | 14.4 | 0.9 | - | - |
| Mai, T., et al. | - | - | - | - | - | - |
| Toyokawa-S., K.C., et al. | - | - | - | - | - | - |

**Supplementary Table 5.** Exclusion criteria for included studies

| **Exclusion criteria** | Chen, H., et al. | Eissa, O., et al. | Li, Y., et al. | Almagrami, I., et al. | Mai, T., et al. | Toyokawa-S., K.C., et al. |
| --- | --- | --- | --- | --- | --- | --- |
| Existing root resorption | x | x | x | x | x | x |
| Dental trauma | x | x | x | x |  | x |
| History of orthodontic treatment | x |  | x | x | x | x |
| Missing teeth | x | x | x | x | x | x |
| History of endodontic treatment | x | x | x | x | x |  |
| Severe crowding | x | x |  | x | x | x |
| Defects, deformities | x |  | x | x |  | x |
| Periodontal disease | x |  | x | x |  |  |
| Treatment with extractions |  |  |  | x | x | x |
| Caries | x |  | x |  |  |  |
| Supernumerary or impacted teeth | x |  | x |  |  |  |
| Temporomandibular disorder | x |  | x |  |  |  |
| Open bite |  | x |  |  |  | x |
| Other systemic conditions |  | x |  | x |  |  |
| Smoking |  |  |  | x |  |  |
| Crossbite |  |  |  |  |  | x |
| Tooth in need of restoration |  |  |  |  |  | x |
| Poor compliance | x |  |  |  |  |  |
| Parafunctions |  | x |  |  |  |  |
| Deep bite |  | x |  |  |  |  |
